# Supplementary material for: EZH2-mediated PP2A inactivation confers resistance to HER2-targeted breast cancer therapy
Source: Nat Commun. 2020 Nov 18;11:5878. doi: 10.1038/s41467-020-19704-x (PMC7674491; doi:10.1038/s41467-020-19704-x)
Supplement: Supplementary file 1 — Supplementary Information [file 41467_2020_19704_MOESM1_ESM.pdf]

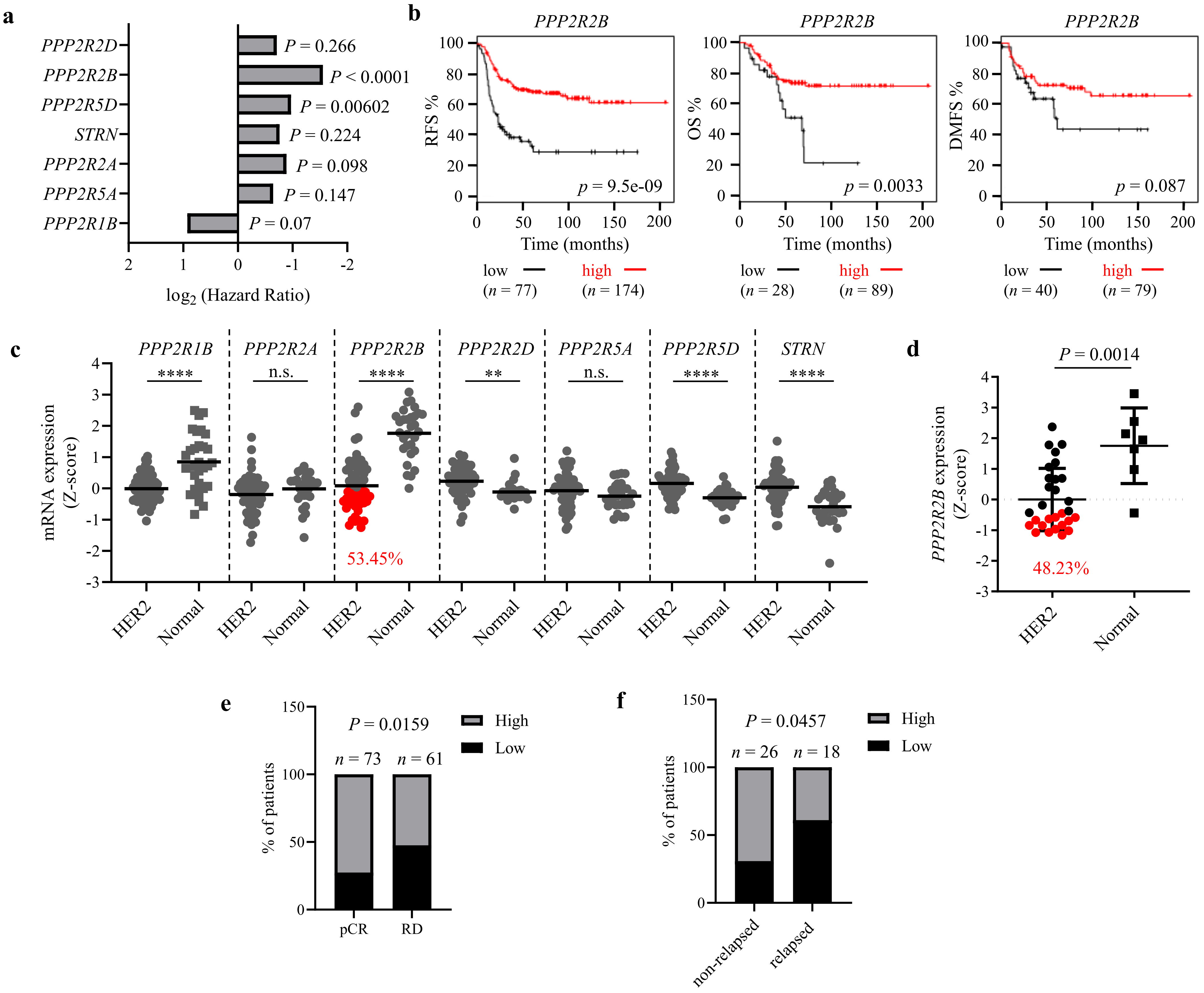

Supplementary Figure 1. Downregulation of *PPP2R2B* signifies poor prognosis of HER2+ breast cancer.

a Hazard ratio (log2) between expression of the indicated PP2A members and relapse-free survival (RFS) in HER2+ breast cancer from KM Plotter. Bonferroni-adjusted *P* values are indicated. **b** Kaplan-Meier curves comparing RFS, overall survival (OS), and DMFS of HER2+ breast cancer patients with tumors expressing high (red) or low (black) levels of *PPP2R2B* from KM Plotter (http://kmplot.com/analysis/). The computed based performing thresholds which are used as cut-offs were auto-selected for the percentiles of the subjects between the low and high gene-expression groups in (a) and (b). *P* values in (a) and (b) were calculated with two-sided log-rank test. **c** Scatterplot showing expression of the indicated PP2A members in HER2+ breast cancer tumors ($n = 58$) compared with normal tissues ($n = 30$). Mean of each group is presented. *P* values were determined with two-sided Student's *t*-test, and corrected with Bonferroni adjustment. n.s., not significant, $^{**}P < 0.01$, $^{****}P < 0.0001$. Tumor samples that express levels of *PPP2R2B* lower than any normal tissues are highlighted in red, and their proportion to all tumors is indicated. **d** Expression of PPP2R2B assessed by qPCR in HER2+ breast cancer tumors ($n = 29$), compared with normal tissues ($n = 7$). The qPCR was performed with pre-amplified cDNA products. Tumor samples that express levels of *PPP2R2B* lower than any normal tissues were highlighted in red, and their proportion to all tumors is indicated. Values represent means ± s.d. *P* value was calculated with two-sided Mann-Whitney *U* test. **e** Two-sided Chi-Square test of the relationship between low expression of *PPP2R2B* and residual disease (RD) after trastuzumab treatment, in HER2+ breast cancer. pCR: pathologic complete response. **f** Two-sided Chi-Square test of the relationship between low expression of *PPP2R2B* and relapse in five years after trastuzumab treatment, in HER2+ breast cancer. High or Low *PPP2R2B* expression in (e) and (f) is defined with a cut-off of a Z-score of -0.5. Data in (e) and (f) are acquired from ROC Plotter (http://www.rocplot.org/).

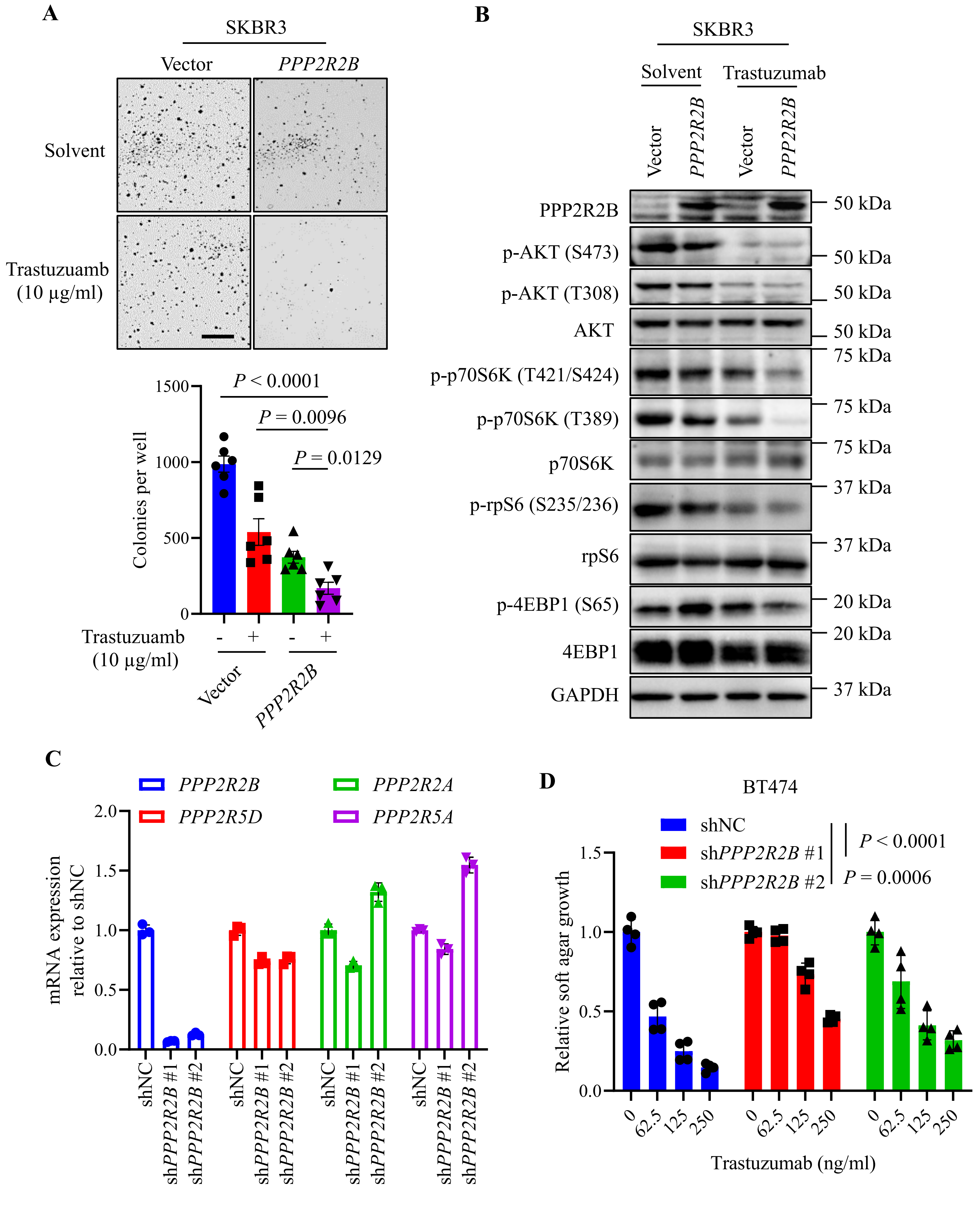

Supplementary Figure 2. Role of *PPP2R2B* in modulating response to HER2-targeted therapies.

a Representative images (top) and quantification (bottom) of soft agar assay with SKBR3 transduced with empty vector (Vector) or *PPP2R2B* overexpression (*PPP2R2B*) and treated with the indicated concentration of trastuzumab. Values represent means ± s.e.m. of two independent overexpression experiments performed in triplicate. *P* values were determined with two-sided Student's *t*-test, and corrected with Bonferroni adjustment. Scale bar: 1.5 mm. **b** Western blot analysis with cells from (a) treated with trastuzuamb (10 µg/ml). **c** RT-qPCR assessing the expression of the indicated PP2A subunits in BT474 received knock-down of *PPP2R2B*. Values represent means ± s.d. of technical triplicates. **d** Soft agar assay with BT474 transduced with shNC or shRNAs against *PPP2R2B* and treated with the indicated concentrations of trastuzuamb. Values represent means ± s.d. of two independent experiments performed in duplicates (n = 4). *P* values were determined by two-way ANOVA, and corrected with Bonferroni adjustment.

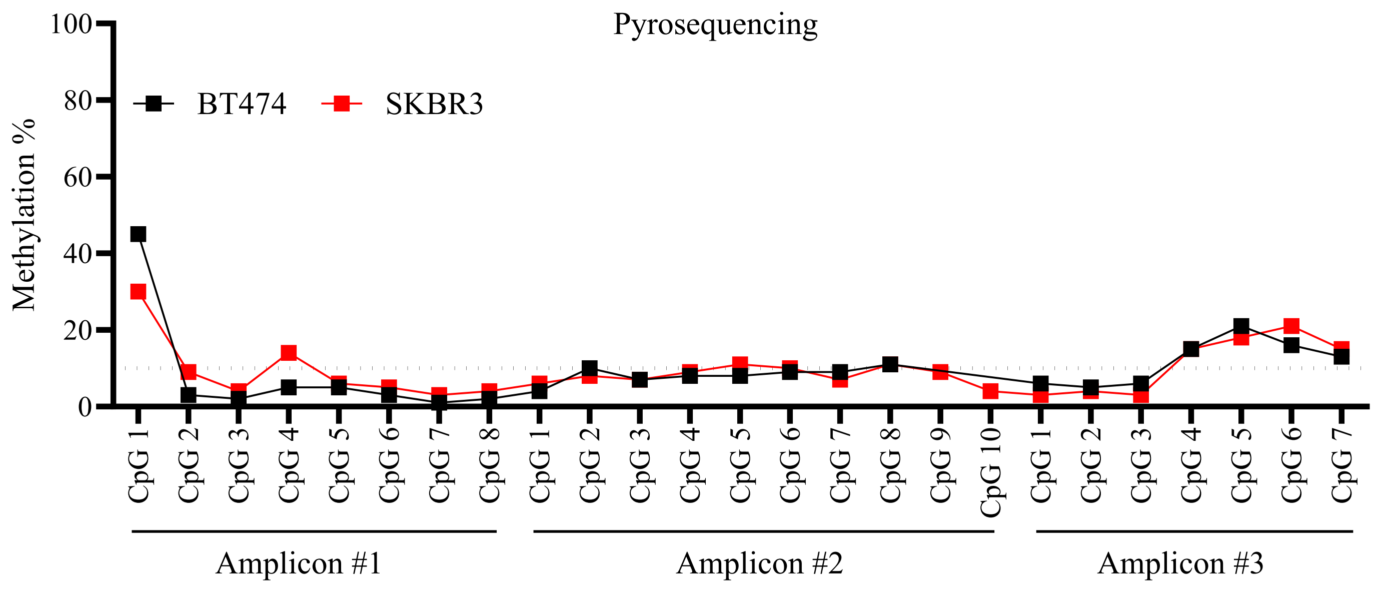

Supplementary Figure 3. Increased DNA methylation is not detected in SKBR3 compared to BT474 at *PPP2R2B* promoter.

Quantitative profiles of DNA methylation levels across multiple CpG sites at *PPP2R2B* promoter in BT474 and SKBR3, using pyrosequencing with three amplicons.

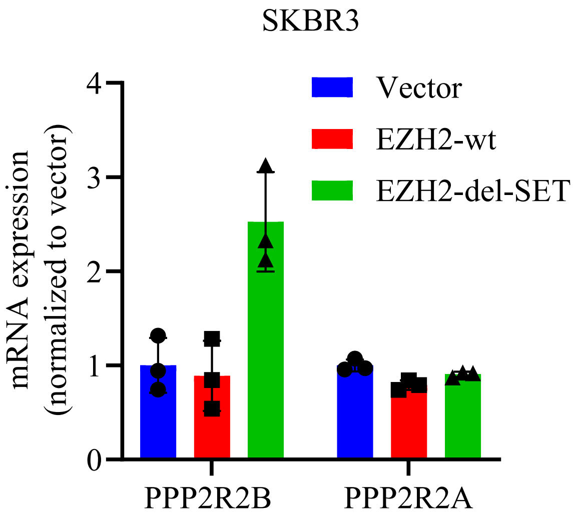

Supplementary Figure 4. Negative dominant EZH2 induces PPP2R2B.

RT-qPCR measuring the expression levels of *PPP2R2B* and *PPP2R2A* in SKBR3 overexpressing wild type EZH2 (EZH2-wt) or EZH2 with deletion of its SET domain (EZH2-del-SET). Data are expressed as mean ± s.d. of technical triplicates.

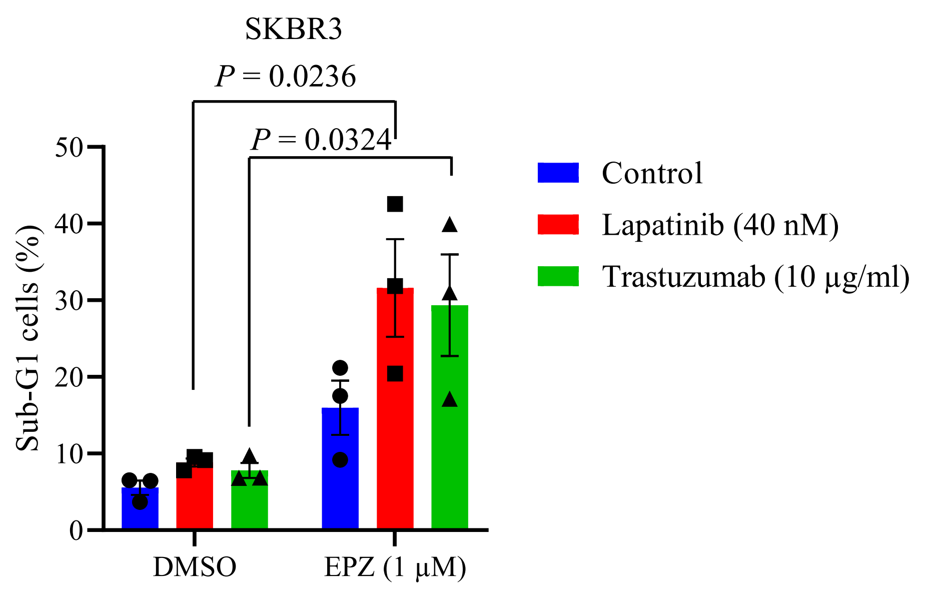

Supplementary Figure 5. Increase of apoptotic cells by cotreatment of EPZ and HER2-targeted therapies in SKBR3.

Quantification of sub-G1 apoptosis assay with SKBR3 cells treated with the indicated compounds at the indicated concentrations. Values represent means ± s.e.m. of three independent experiments. *P* values were determined with two-sided Student's *t*-test.

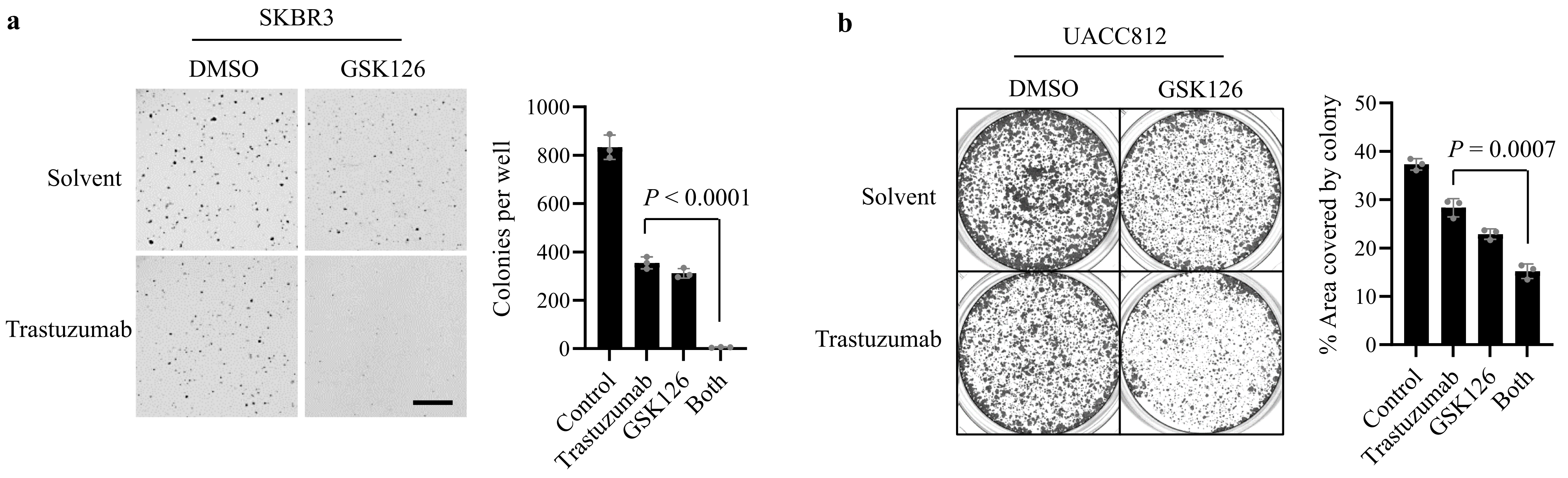

Supplementary Figure 6. GSK126 and trastuzumab combination elicits robust effect *in vitro*.

a Representative images (left) and quantification (right) of soft agar assay with SKBR3 treated with DMSO, GSK126 (1 µM), trastuzumab (10 µg/ml), or the combination of both. Data are expressed as mean ± s.d. of technical triplicates. Scale bar for the left panel: 1.5 mm. **b** Representative images (left) and quantification (right) of clonogenic assay with UACC812 treated with DMSO, GSK126 (1 µM), trastuzumab (10 µg/ml), or the combination of both. Data are expressed as mean ± s.d. of technical triplicates. *P* values in (A) and (B) were determined with two-sided Student's *t*-test.

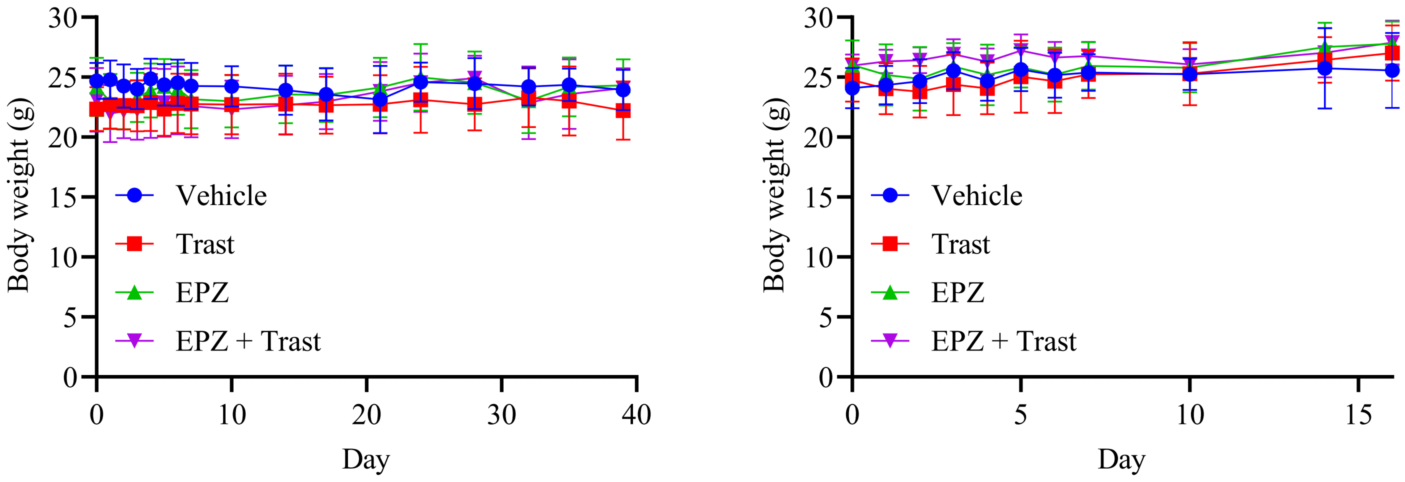

Supplementary Figure 7. Body weight of the mice under treatment.

The body weight of mice bearing UACC812 (left) or MB361 (right) xenografts treated in Figure 6, A and B. Mice with UACC812 xenografts were treated with vehicle (*n* = 7), EPZ (*n* = 8), trastuzumab (trast; *n* = 8), or in combination (EPZ + Trast; *n* = 7). Mice with MB361 xenografts were treated with vehicle (*n* = 7), EPZ (*n* = 6), trastuzumab (trast; *n* = 6), or in combination (EPZ + Trast; *n* = 6). Values represent means ± s.d.

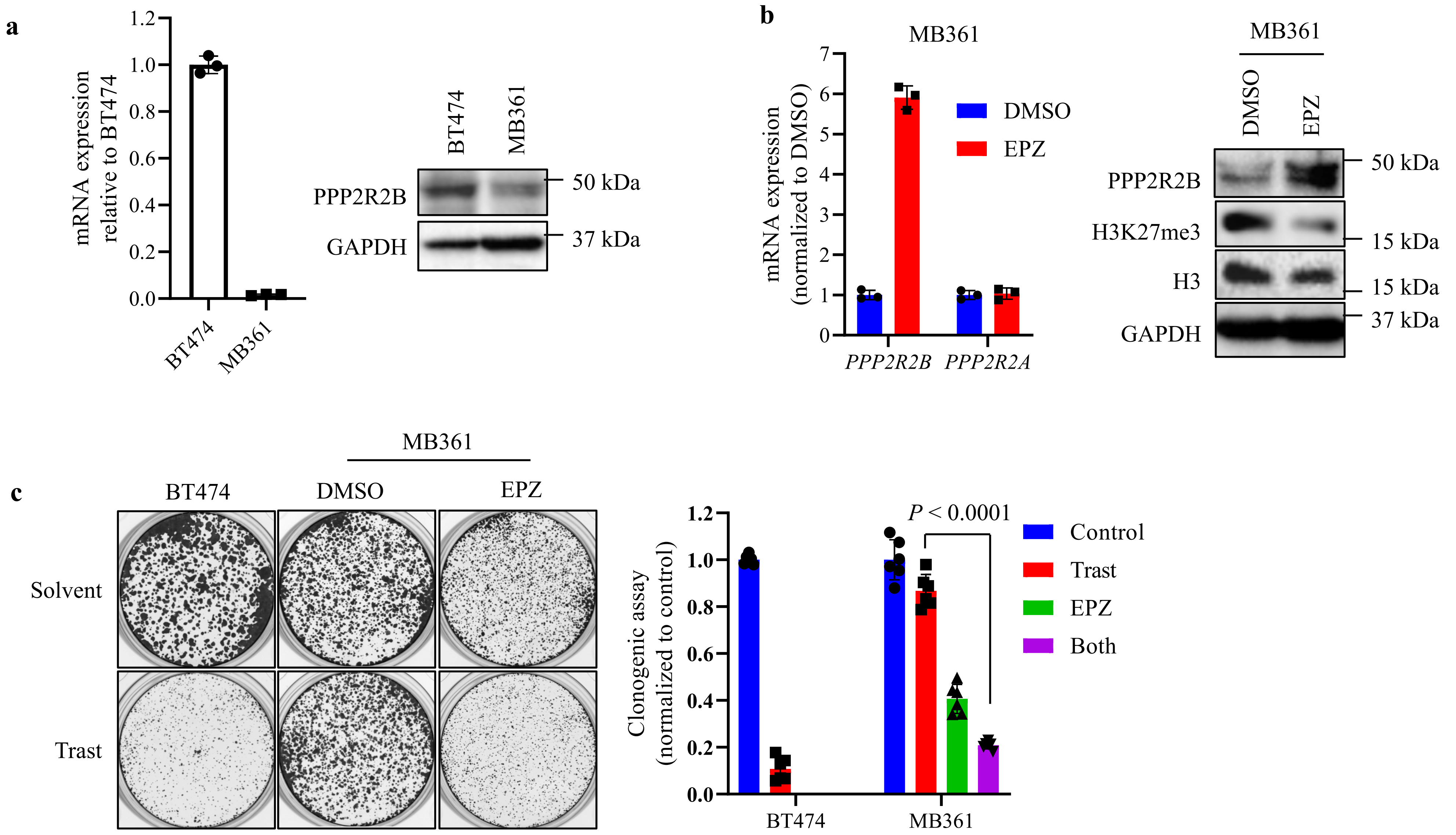

Supplementary Figure 8. EPZ mitigate trastuzumab resistance in MB361.

a RT-qPCR (left) and western blot (right) assessing the expression of *PPP2R2B* in the indicated cell lines. **b** RT-qPCR (left) and western blot (right) assessing the expression of *PPP2R2B* in MB361 treatment with DMSO or EPZ (1 μM). RT-qPCR data (a) and (b) are expressed as mean ± s.d. of technical triplicates. **c** Representative images (left) and quantification (right) of clonogenic assay with cells treated with the indicated compounds. EPZ was given at 1 μM and trastuzumab (trast) at 10 μg/ml . Data are expressed as mean ± s.d. of two independent experiments performed in triplicate (n = 6). P value was determined with two-tailed Student's t-test.

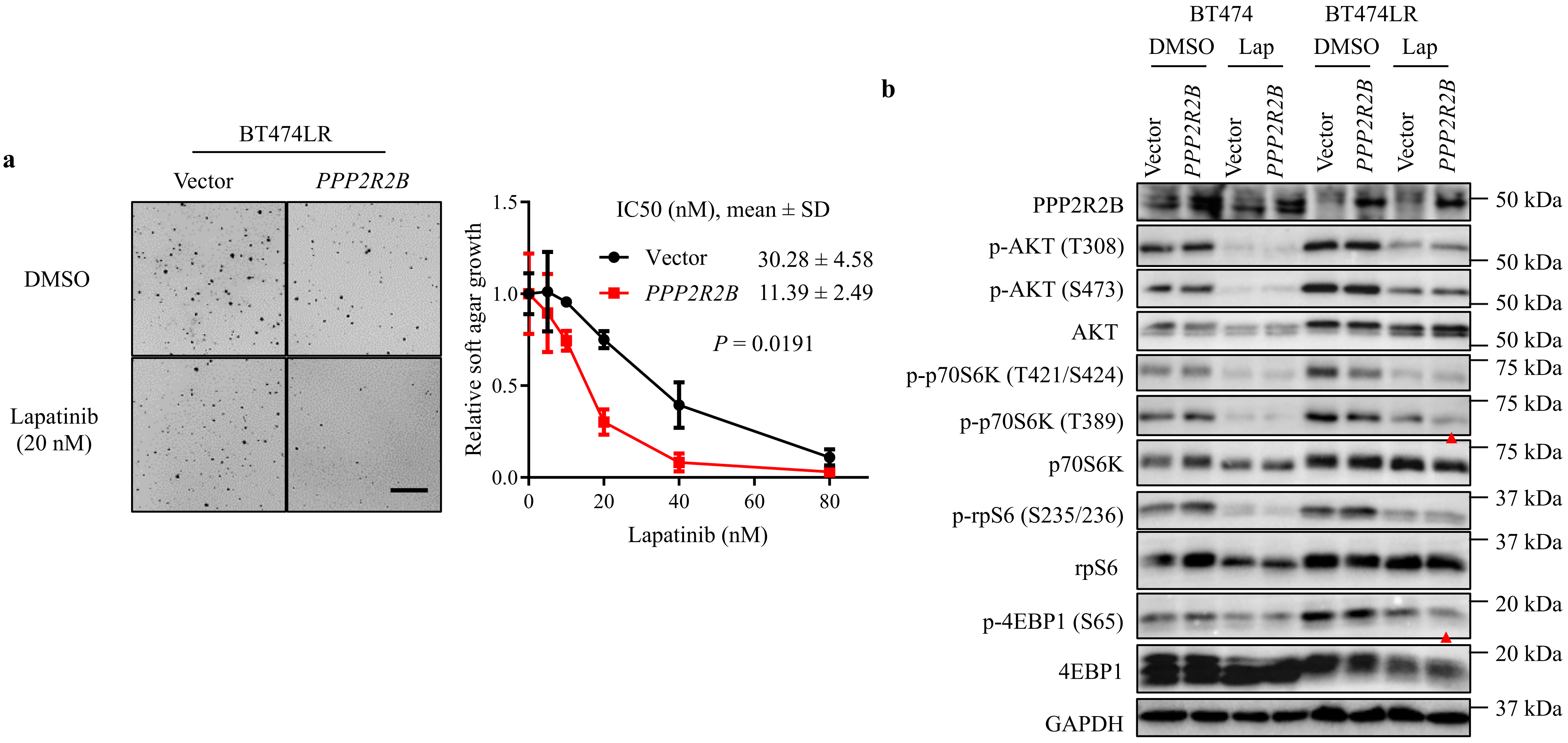

Supplementary Figure 9. Ectopic PPP2R2B sensitizes BT474LR and suppresses phosphorylation of p70S6K and 4EBP1 under lapatinib treatment.

a Representative images (left) and quantification (right) of soft agar assay with BT474LR transduced with the empty vector (Vector) or the vector carrying *PPP2R2B*, and treated with the indicated concentrations of lapatinib. Data are expressed as means ± s.d and representative of three independent overexpression experiments. The indicated IC50s and *P* value were calculated with the three independent experiments. Scale bar: 1.5 mm. *P* value was determined with two-sided Student's *t*-test. **b** Representative western blot analysis (n = 2) with cells from (A) treated with lapatinib (Lap) at 40 nM. Reduction of phosphorylation downstream of mTOR in the resistant cells is highlighted in red.

Supplementary Figure 10. Uncropped western blots.

Uncropped Western blots for Fig. 2a

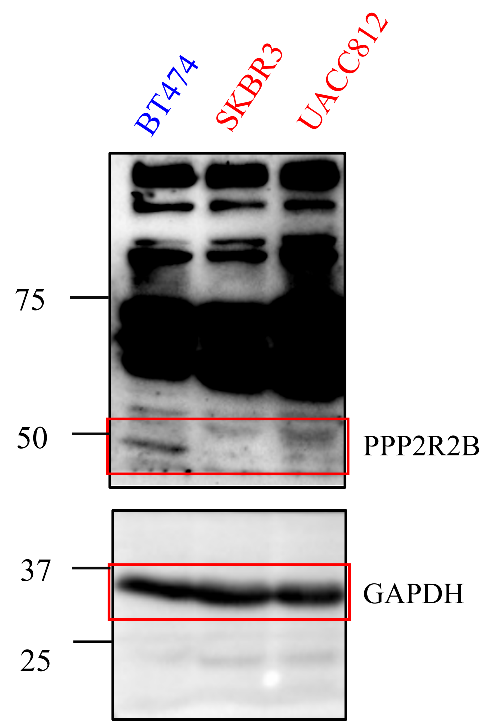

Uncropped Western blots for Fig. 2d

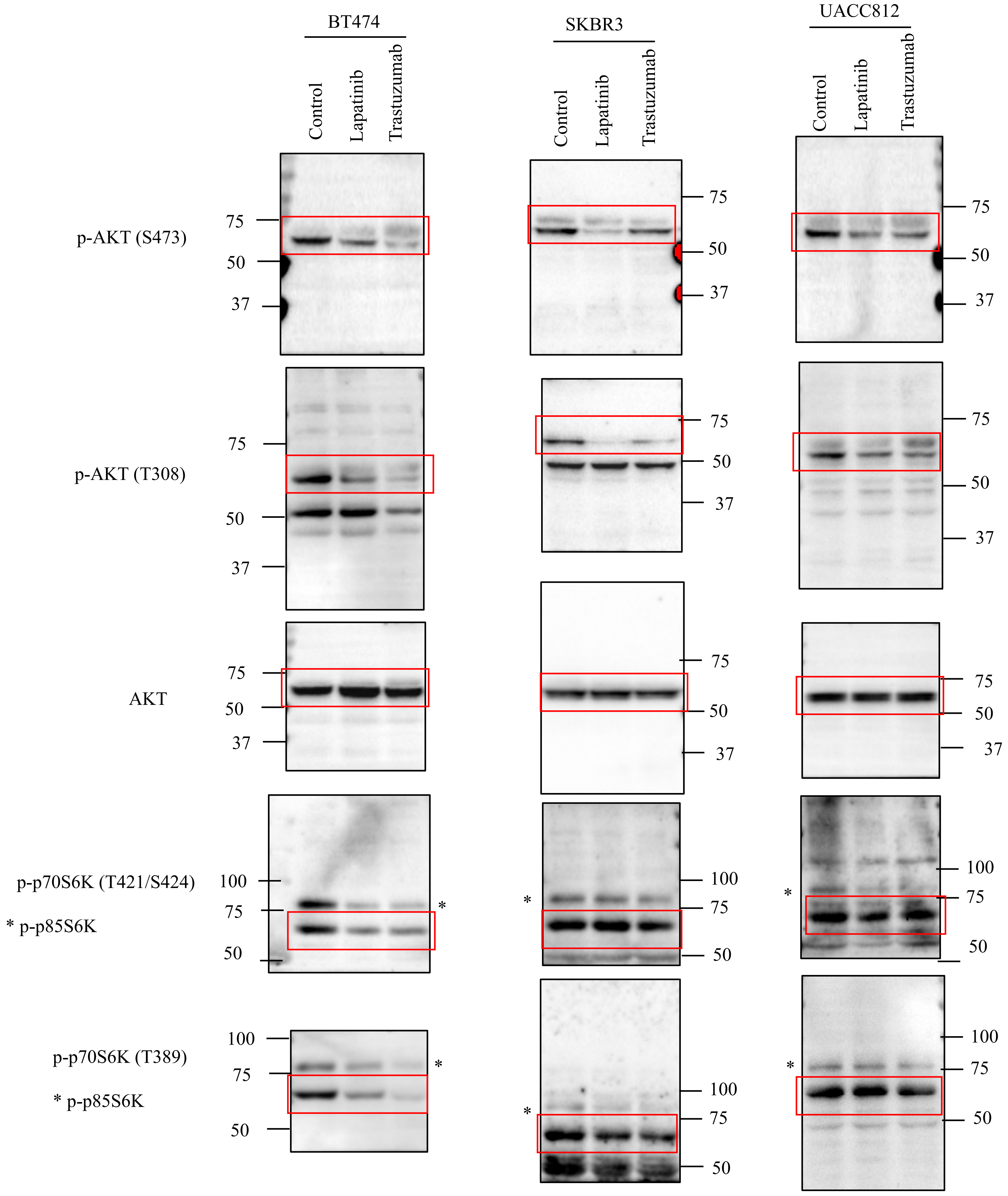

Uncropped Western blots for Fig. 2d (continued)

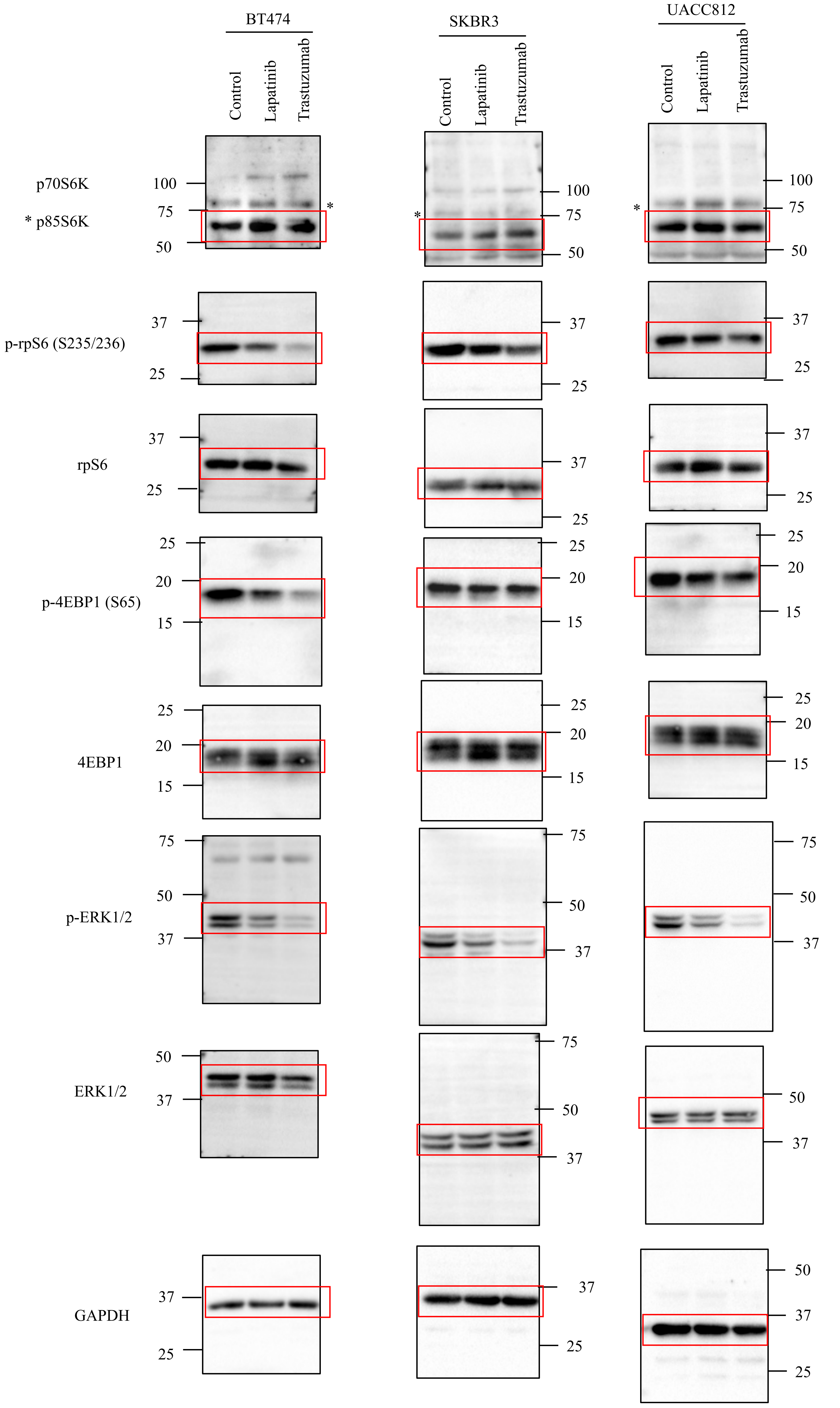

Uncropped Western blots for Fig. 3c

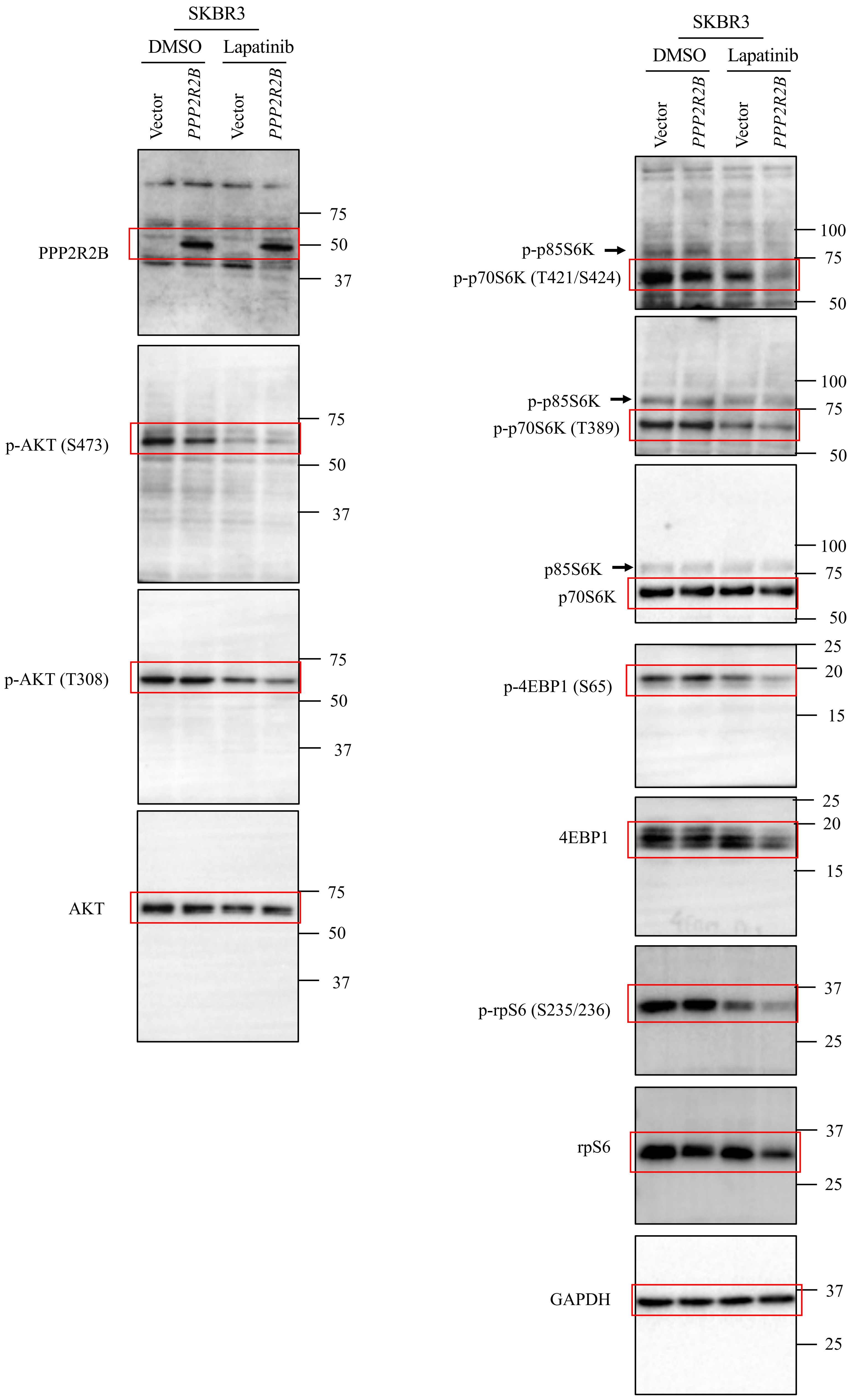

Uncropped western blots for Supplementary Fig. 2b

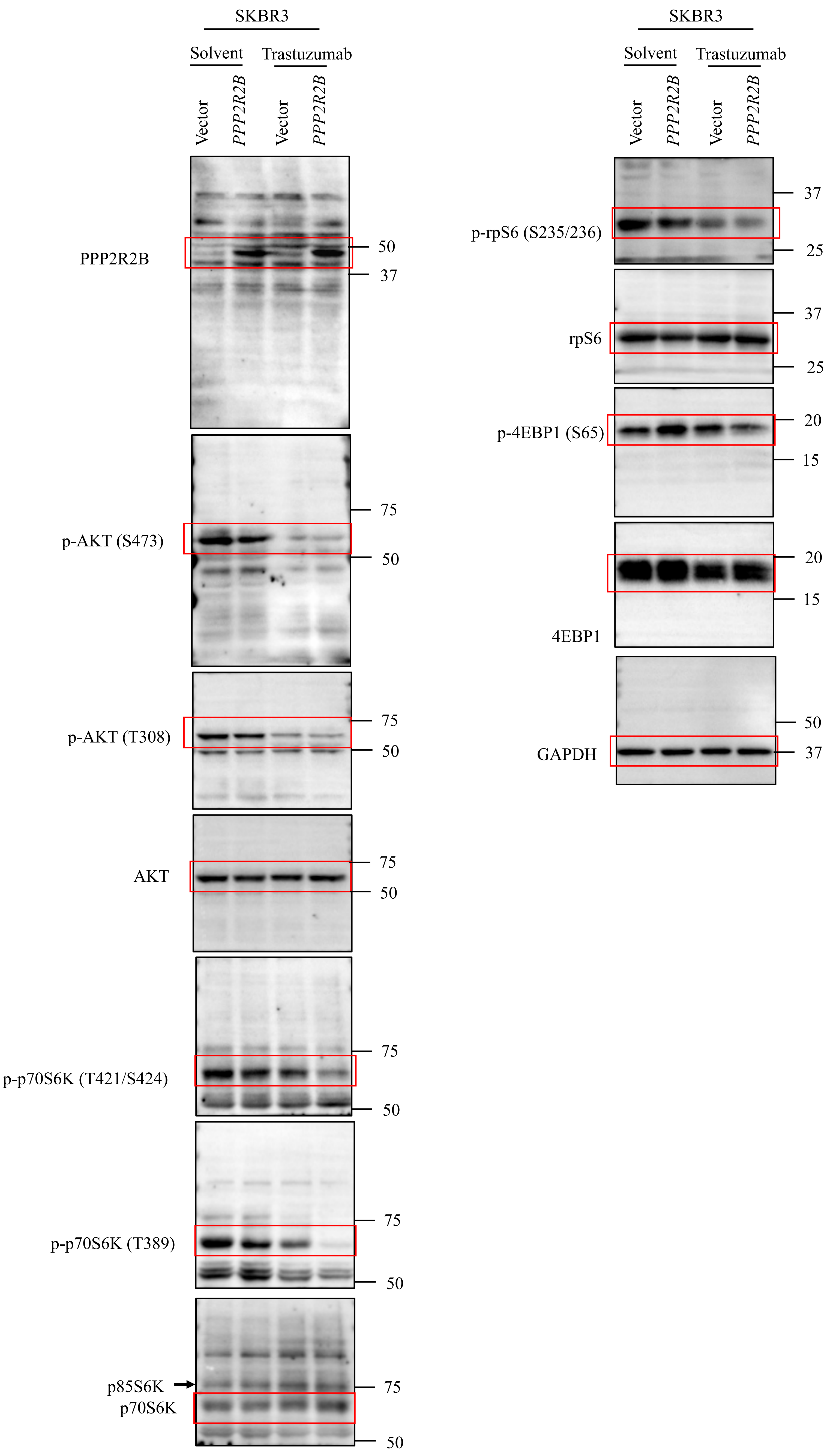

Uncropped Western blots for Fig. 3d

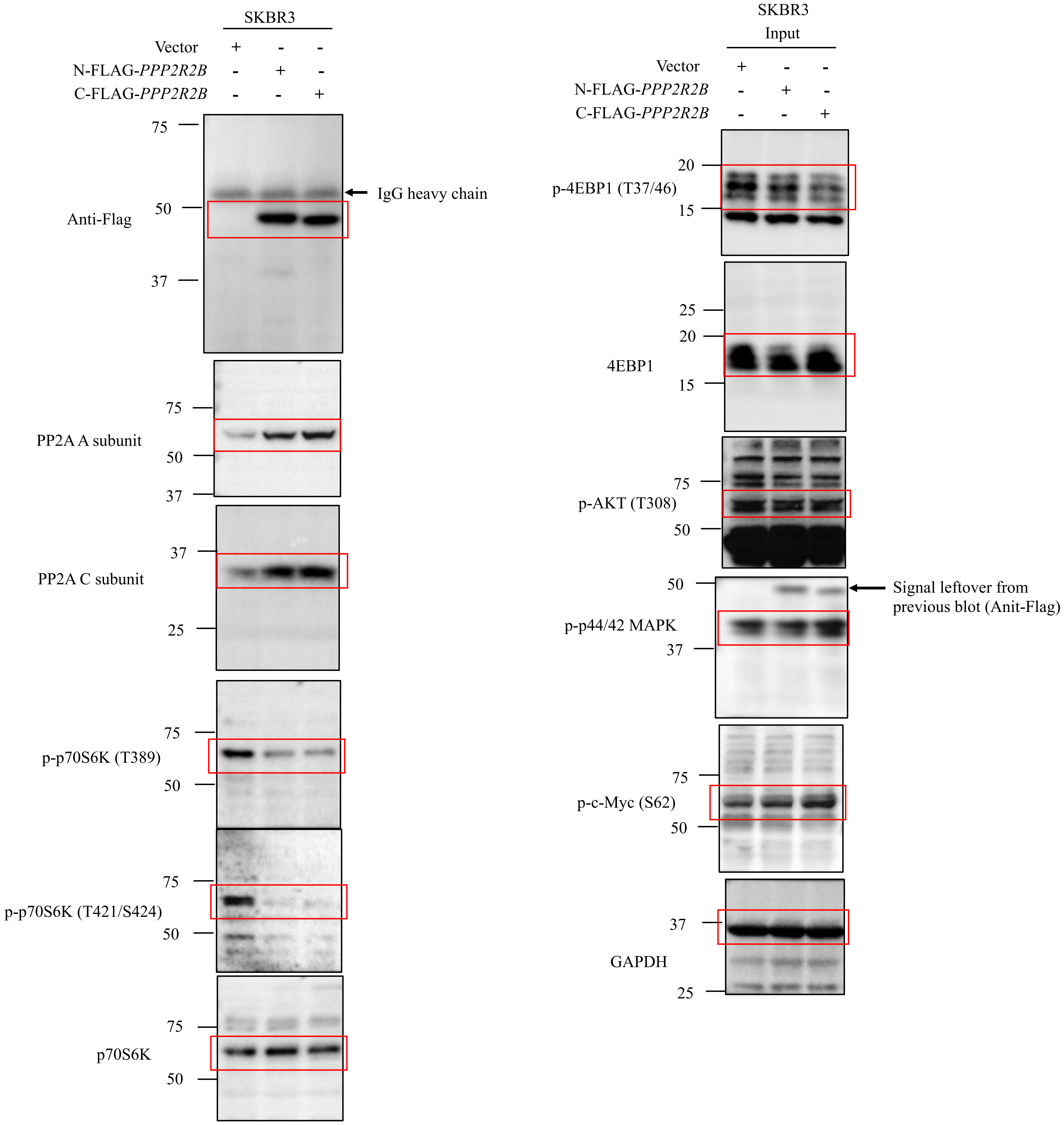

Uncropped Western blots for Fig. 3f

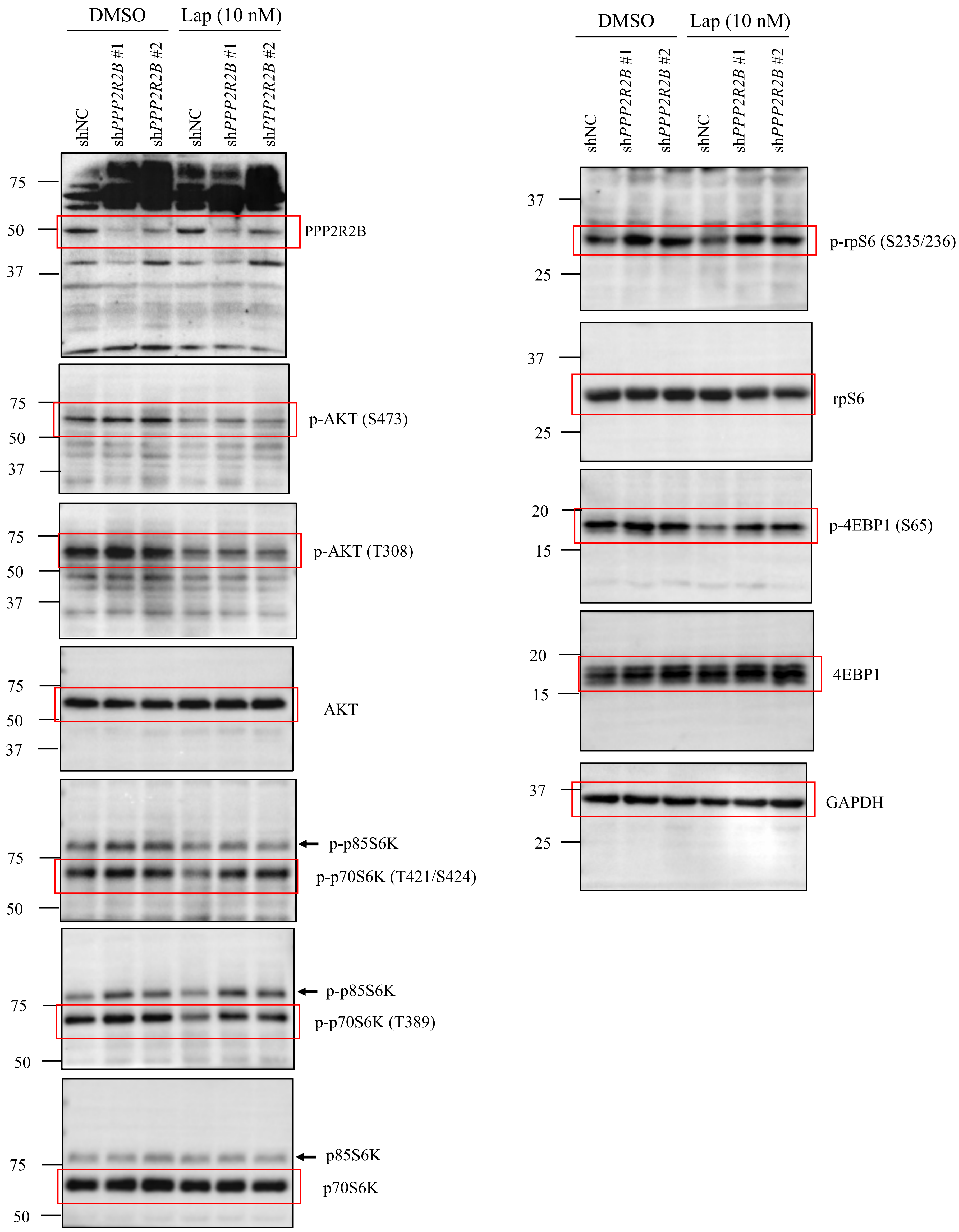

Uncropped Western blots for Fig. 4b

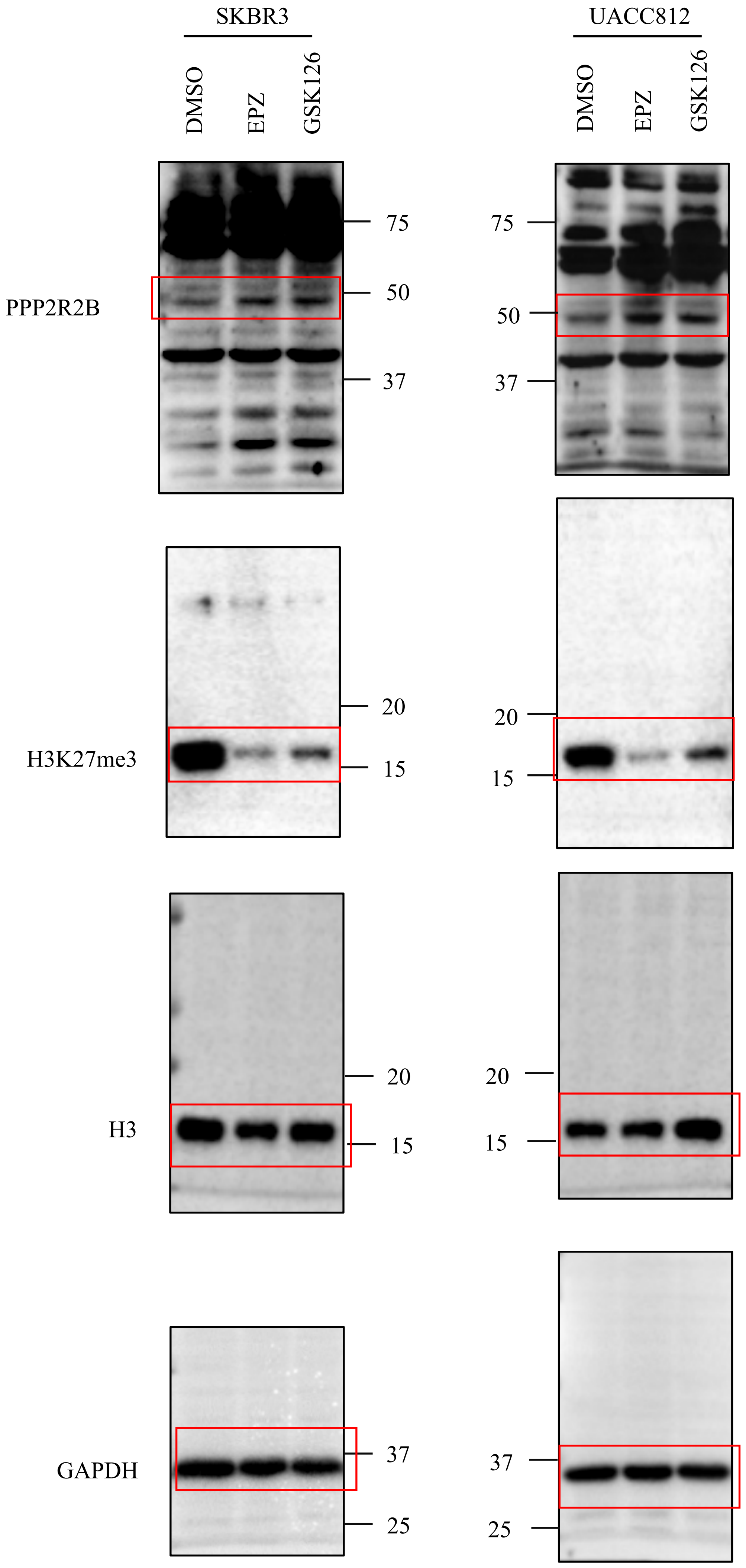

Uncropped Western blots for Fig. 4e

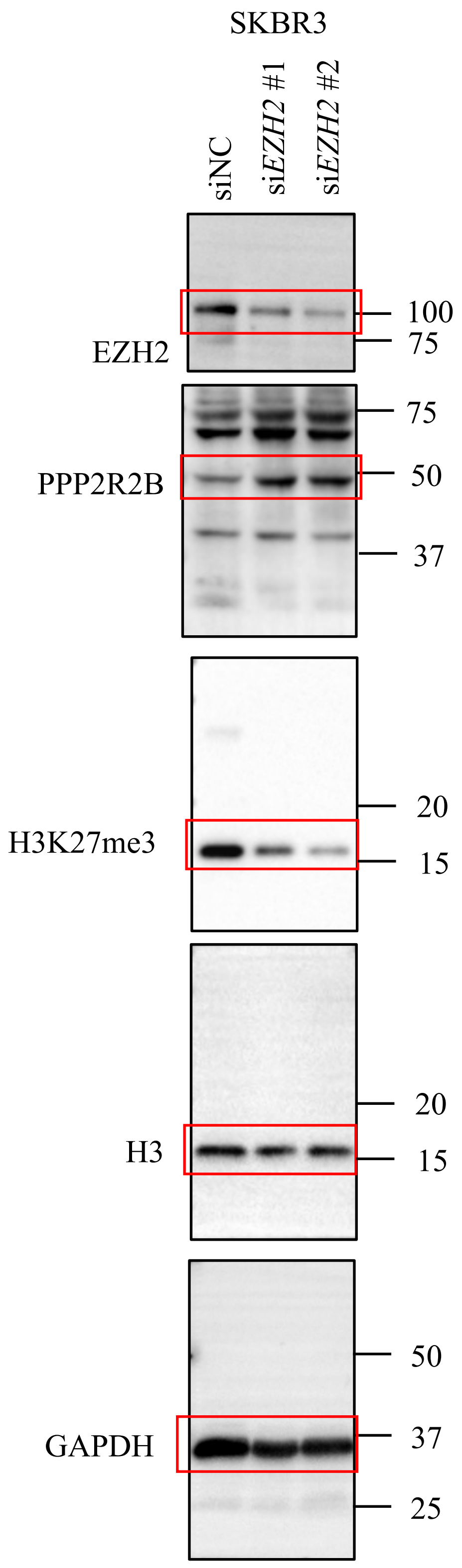

Uncropped western blots for Fig. 4f

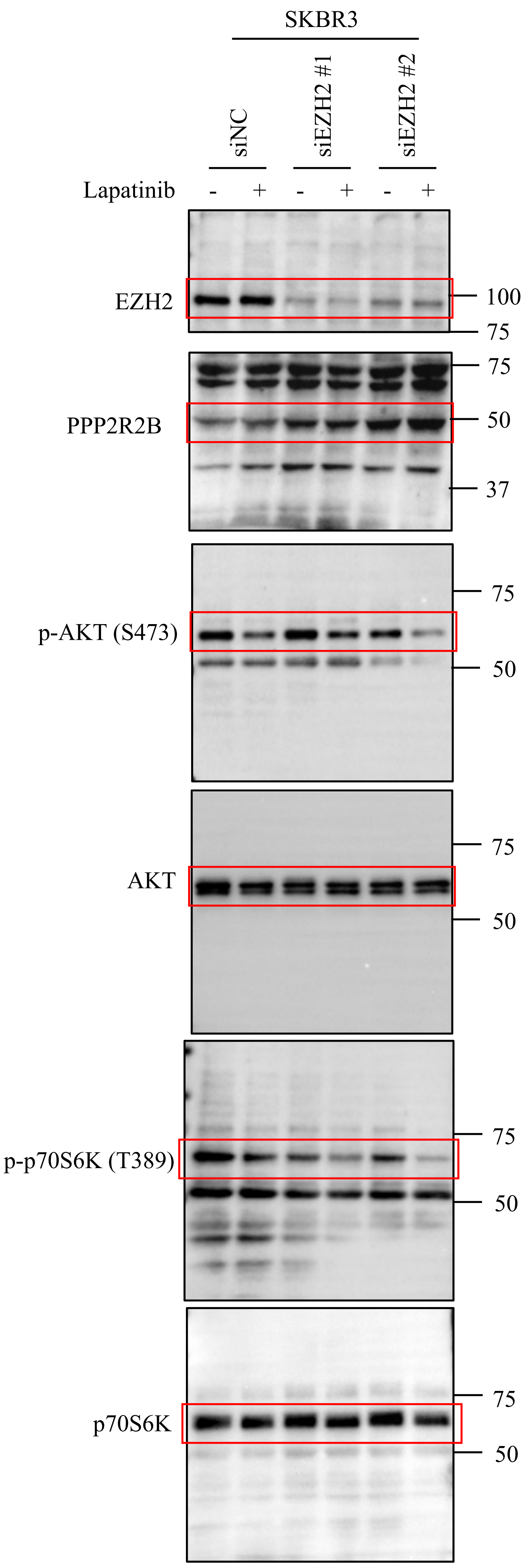

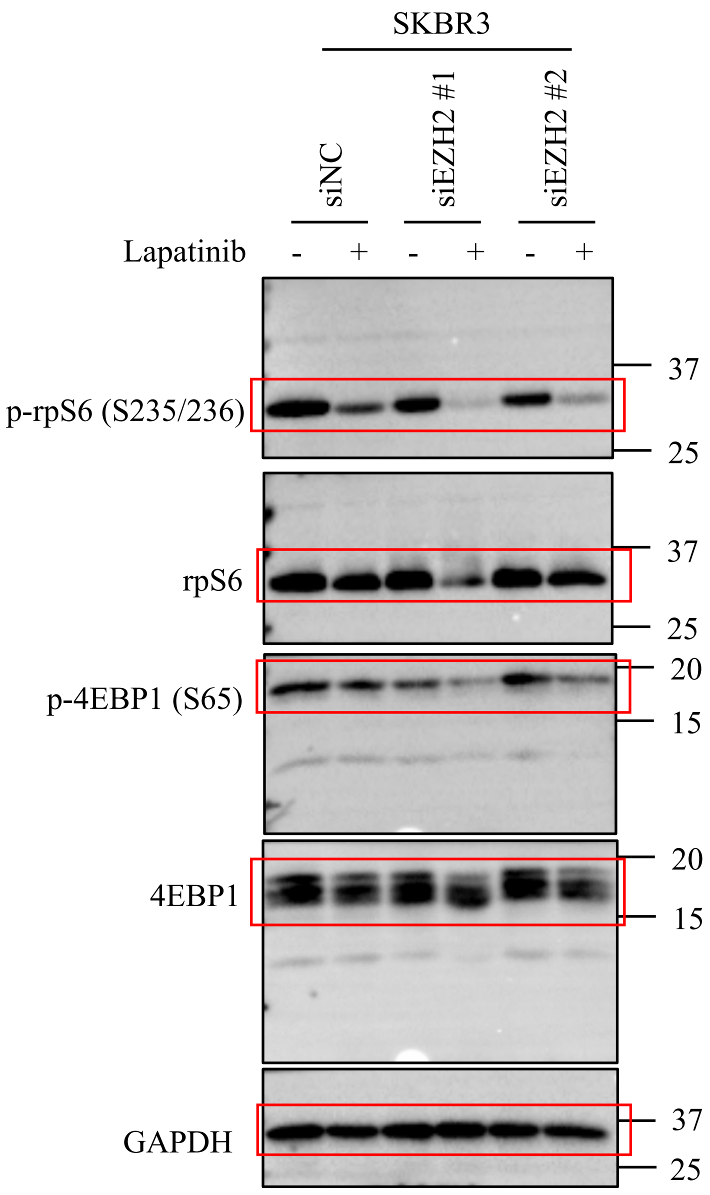

Uncropped Western blots for Fig. 5c

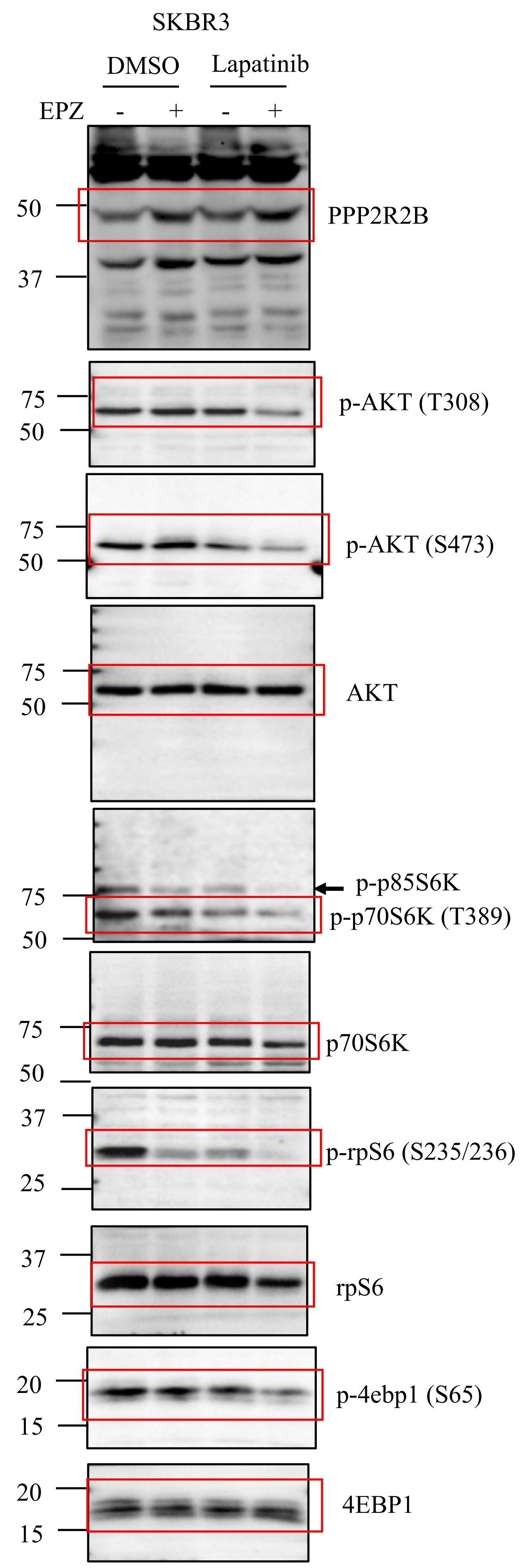

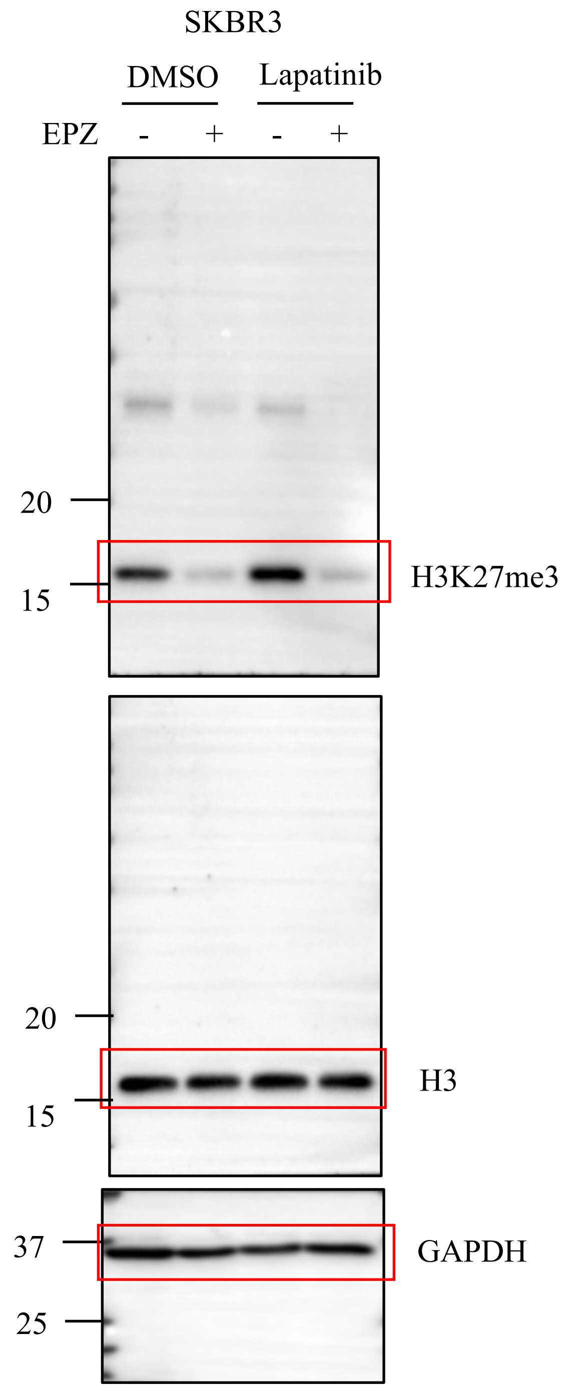

Uncropped western blots for Fig. 5d

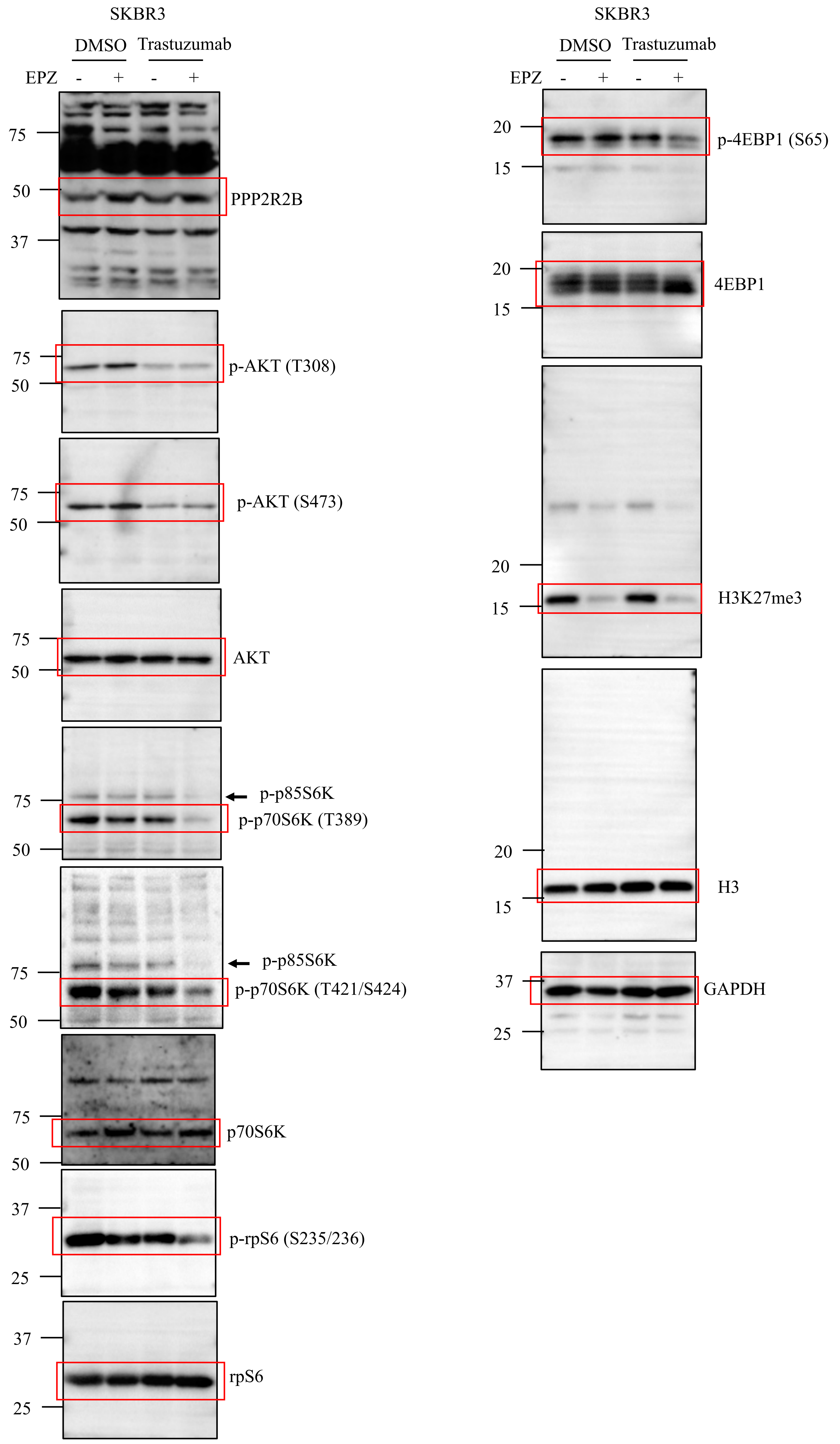

Uncropped western blots for Fig. 7e

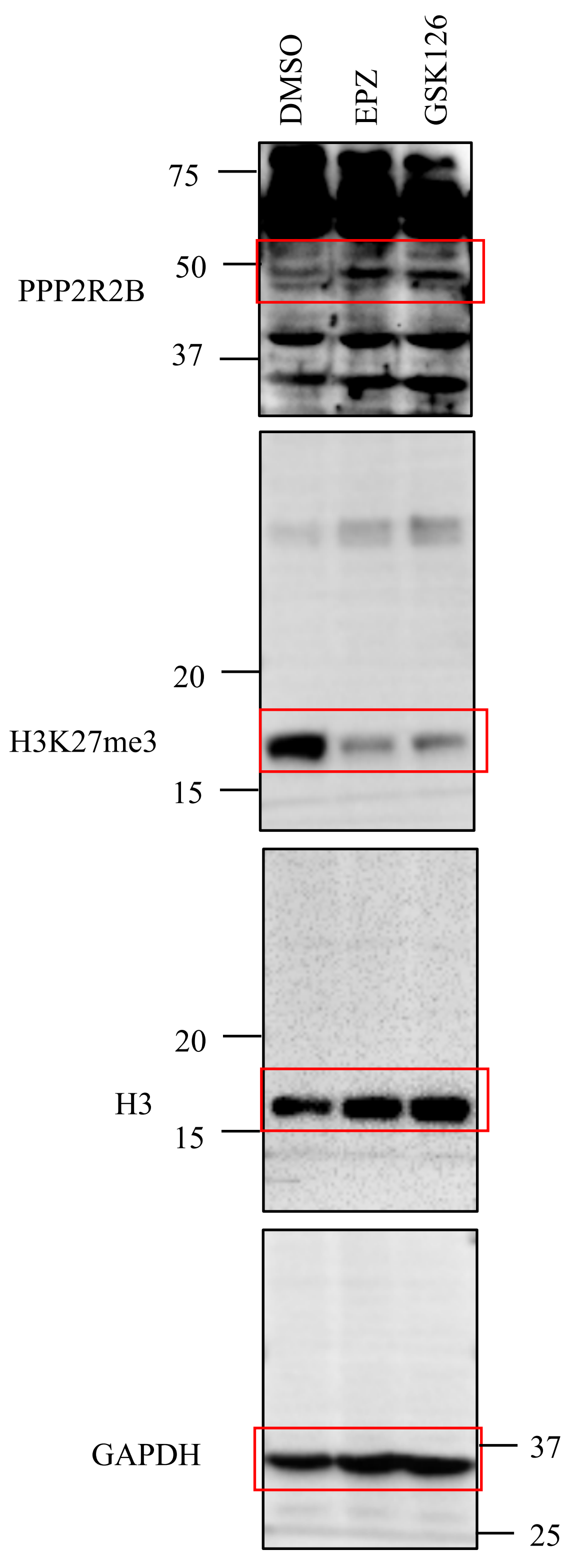

Uncropped western blots for Supplementary Fig. 8a

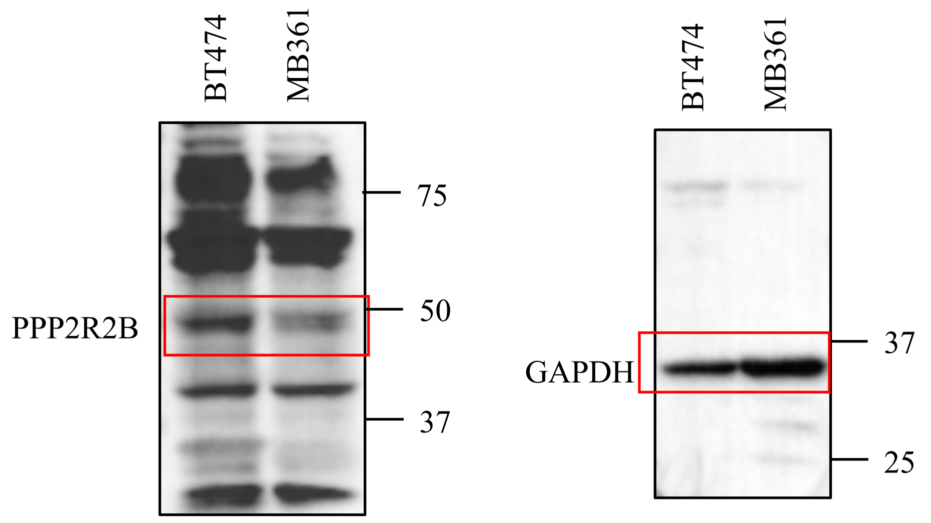

Uncropped western blots for Supplementary Fig. 8b

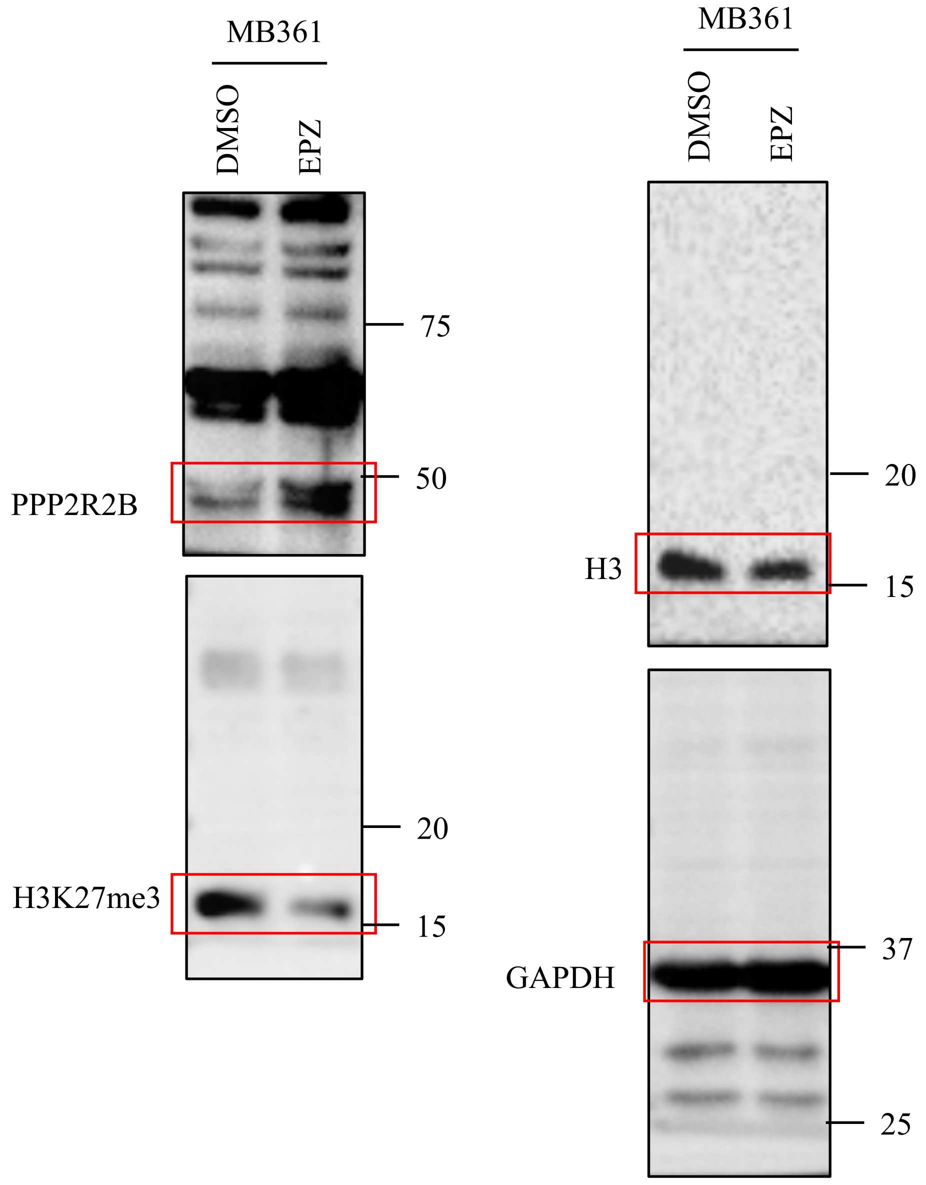

Uncropped western blots for Supplementary Fig. 9

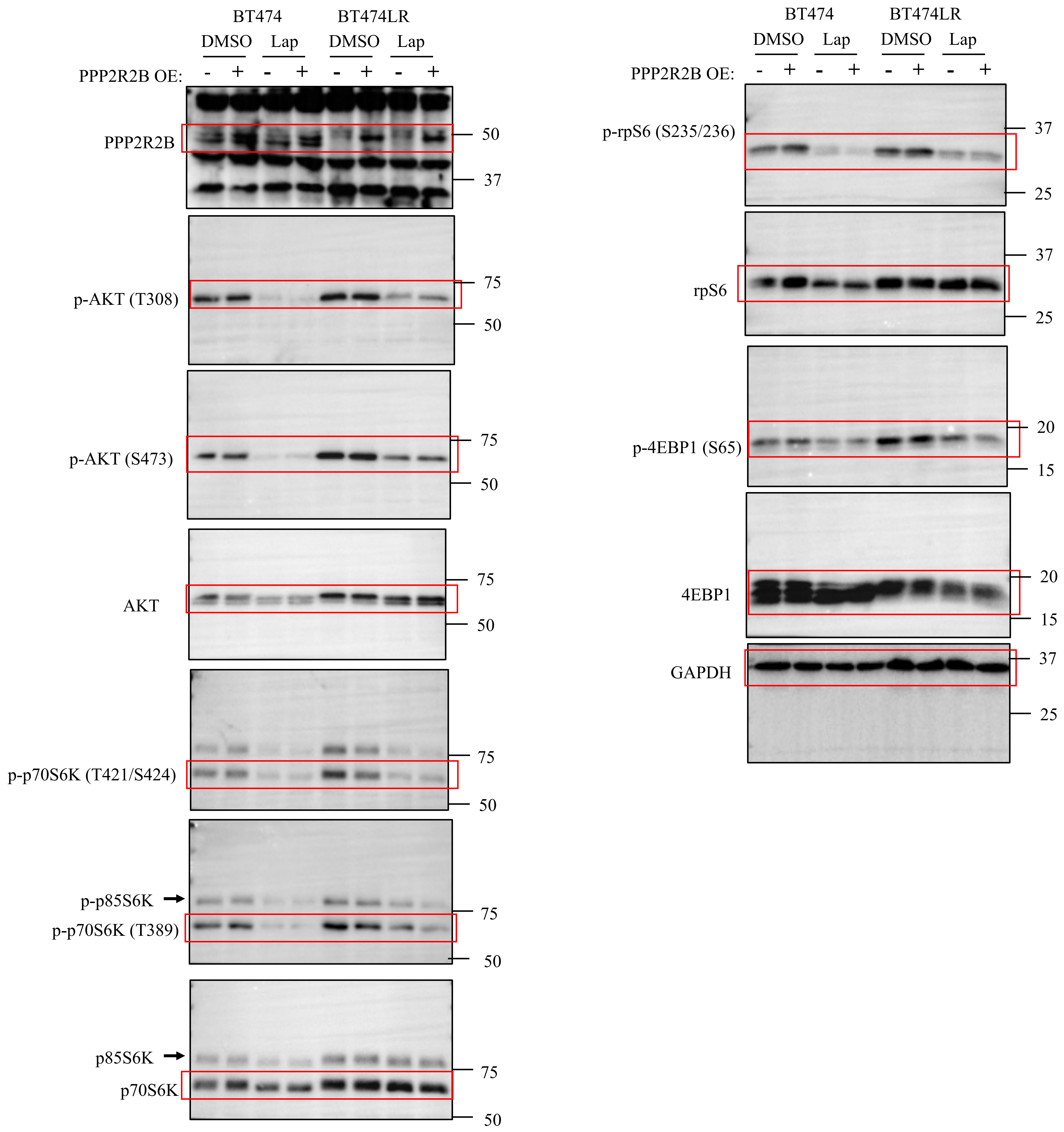

Supplementary Table 1. Hazard ratios and P values of 18 known PP2A subunit gene expression in relapse-free survival of breast cancer patients.

Expression of 18 known PP2A subunit genes was analyzed about relapse-free survival (RFS) in breast cancer, with KM Plotter (http://kmplot.com/analysis/). The hazard ratio and *P* value for each subunit gene are listed in the table. A hazard ratio lower than one indicates that low expression of the subunit is associated with poor RFS. The adjusted *P* values are acquired with a Bonferroni test. Subunit genes with hazard ratio lower than one and adjusted P value lower than 0.05 are considered candidate subunits and highlighted in red. *P* values were determined with two-sided log-rank test.

Gene Name	Probe	Hazard Ratio	*P* value	Adjusted *P* value	*n* number
PPP2R1A	200695_at	0.85	0.0047	0.0846	3951
PPP2R1B	222351_at	0.77	< 0.0001	0.0006	3951
PPP2R2A	228013_at	0.61	< 0.0001	< 0.0001	1746
PPP2R2B	213849_s_at	0.55	< 0.0001	< 0.0001	3951
PPP2R2C	228010_at	0.86	0.084	1	1746
PPP2R2D	225066_at	0.5	< 0.0001	< 0.0001	1746
PPP2R5A	202187_s_at	0.77	< 0.0001	0.0003	3951
PPP2R5B	204611_s_at	1.16	0.025	0.45	3951
PPP2R5C	201877_s_at	0.95	0.32	1	3951
PPP2R5D	202513_s_at	0.6	< 0.0001	< 0.0001	3951
PPP2R5E	229322_at	1.64	< 0.0001	< 0.0001	1746
PPP2R3A	209633_at	1.25	0.0001	0.0017	3951
PPP2R3B	219264_s_at	1.49	< 0.0001	< 0.0001	3951
PPP2R3C	218852_at	1.46	< 0.0001	< 0.0001	3951
STRN	236980_at	0.58	< 0.0001	< 0.0001	1746
STRN3	204496_at	1.24	0.001	0.018	3951
PPP2CA	208652_at	1.17	0.006	0.108	3951
PPP2CB	201375_s_at	0.72	0.17	1	3951

Supplement Table 2. List of gene-specific primers used for qPCR.

Primers	Sequence (5' to 3')	Size of amplicon
GAPDH-F	GGAGCGAGATCCCTCCAA	102 bp
GAPDH-R	AAATGAGCCCCAGCCTTC	
PPP2R2A-F	TGCAGATGATTTGCGGATTA	127 bp
PPP2R2A-F	TGGATGAAATTCTGCTGCTG	
PPP2R2B-F	TGCAGCTTACTTTCTTCTGTCT	85 bp
PPP2R2B-R	GTAGCCTTCTGGCCTCTTATC	
PPP2R5A-F	TGATCCAGAAGAGGATGAACC	111 bp
PPP2R5A-F	GCAATGCTAGGCTGGAAATC	
PPP2R5D-F	GTGGAGGCGAGAACACTGAG	103 bp
PPP2R5D-R	CTATTGCTGGGACGCTTGTT	
EZH2-F	CGCTTTTCTGTAGGCGATGT	84 bp
EZH2-R	AAGTGTTGGGTGTTGCATGA	

Supplement Table 3. List of siRNAs and shRNAs.

siRNA or shRNAs	Targeted Sequence
si*EZH2* #1	5’-CGGTGGGACTCAGAAGGCA-3′
si*EZH2* #2	5’-GTGTTCGGTGACCAGTGACTT-3′
shNC	5’-GTTACGCTGAGTACTTCGA-3’
sh*PPP2R2B* #1	5’-GAAAGTCAGCGAGCGTGATAA-3’
sh*PPP2R2B* #2	5’-GACTTACCAGGTTCATGACTA-3′
sh*EZH2* #1	5’- CGGTGGGACTCAGAAGGCA-3′
sh*EZH2* #2	5’- GGTGAATGCCCTTGGTCAATA-3′
sh*EZH2* #3	5’- GCGCACTTCCTCCTGAATGTA-3′

Supplement Table 4. List of primers used for ChIP-qPCR.

	ChIP Forward Primer 5'--> 3'	ChIP Reverse Primer 5'-->3'
PPP2R2B #1	TGTCCAAAACGAAGTGCAAA	ACACAGGTGGAGGAAAGCAC
PPP2R2B #2	GTAGACGTGGCCCTTAGCTG	AGCGACTAGCTTGCAGGTTC
PPP2R2B #3	AGAAAGGCACCATTTTGTCG	GGAGATGCCCAACAGGTTC
PPP2R2B #4	TACCCGCAAAATCAACAACA	AAGCACAGTGATCCGCAACT
ACTB	CCGAAAGTTGCCTTTTATGG	CAAAGGCGAGGCTCTGTG

Supplement Table 5. List of primers used in bisulfite sequencing.

Amplicon	Primer	Primer sequence (5' to 3')	Amplicon size (bp)
#1	Forward	GGTTGTTAAGGAGGGGAATTT	243
	Reverse	Biot-AATAAAAACCCCACAATAAAACTATCACCT	
	Sequencing	GGGGTAAGGAGTATTTTGGTAT	
#2	Forward	Biot-TGTTGTTAATGGAGGAGGATATTG	154
	Reverse	CCAACTACCCAAAAAACACAATAAT	
	Sequencing	CCCAAAAAACACAATAATCC	
#3	Forward	TTGAGGATTATTGTGTTTTTTGGGTAGTTG	202
	Reverse	Biot-ACCTAATCCCTAAAAACCATTTTAAC	
	Sequencing	TTTTGGGTAGTTGGG	
