# Supplementary material for: EZH2-mediated PP2A inactivation confers resistance to HER2-targeted breast cancer therapy
Source: Nat Commun. 2020 Nov 18;11:5878. doi: 10.1038/s41467-020-19704-x (PMC7674491; doi:10.1038/s41467-020-19704-x)
Supplement: Supplementary file 3 — Reporting Summary [file 41467_2020_19704_MOESM3_ESM.pdf]

nature research

Corresponding author(s): Qiang Yu
Last updated by author(s): Oct 14, 2020

Reporting Summary

Nature Research wishes to improve the reproducibility of the work that we publish. This form provides structure for consistency and transparency in reporting. For further information on Nature Research policies, see our Editorial Policies and the Editorial Policy Checklist.

Statistics

For all statistical analyses, confirm that the following items are present in the figure legend, table legend, main text, or Methods section.

n/a	Confirmed	
☐	☒	The exact sample size (*n*) for each experimental group/condition, given as a discrete number and unit of measurement
☐	☒	A statement on whether measurements were taken from distinct samples or whether the same sample was measured repeatedly
☐	☒	The statistical test(s) used AND whether they are one- or two-sided<br>*Only common tests should be described solely by name; describe more complex techniques in the Methods section.*
☒	☐	A description of all covariates tested
☐	☒	A description of any assumptions or corrections, such as tests of normality and adjustment for multiple comparisons
☐	☒	A full description of the statistical parameters including central tendency (e.g. means) or other basic estimates (e.g. regression coefficient) AND variation (e.g. standard deviation) or associated estimates of uncertainty (e.g. confidence intervals)
☐	☒	For null hypothesis testing, the test statistic (e.g. *F*, *t*, *r*) with confidence intervals, effect sizes, degrees of freedom and *P* value noted<br>*Give P values as exact values whenever suitable.*
☒	☐	For Bayesian analysis, information on the choice of priors and Markov chain Monte Carlo settings
☒	☐	For hierarchical and complex designs, identification of the appropriate level for tests and full reporting of outcomes
☐	☒	Estimates of effect sizes (e.g. Cohen's *d*, Pearson's *r*), indicating how they were calculated

Our web collection on statistics for biologists contains articles on many of the points above.

Software and code

Policy information about availability of computer code

Data collection	GelCount 1.2.1.0 (Oxford Optronix), PerkinElmer Harmony 4.8 (Opera Phenix High-Content Screening System), Pryomark Q24 Software 2.0, CompuSyn 1.0, PyroMark Assay Design SW 2.0, and ImageJ 1.51u.
Data analysis	GraphPad Prism version 8

For manuscripts utilizing custom algorithms or software that are central to the research but not yet described in published literature, software must be made available to editors and reviewers. We strongly encourage code deposition in a community repository (e.g. GitHub). See the Nature Research guidelines for submitting code & software for further information.

Data

Policy information about availability of data

All manuscripts must include a data availability statement. This statement should provide the following information, where applicable:

- Accession codes, unique identifiers, or web links for publicly available datasets
- A list of figures that have associated raw data
- A description of any restrictions on data availability

The source data underlying Figs 1A, B, D-F, 2A-C, 3B, E, 4A, C-E, G, 5A, B, E, 6A-C, 7A-G, 8A-F, and Supplementary Figs. 1C-F, 2A, C, D, 3-5, 6A, B, 7, 8A-C, and 9A are provided as a Source Data file. The online dataset GSE62327 is available on GEO database (https://www.ncbi.nlm.nih.gov/geo/query/acc.cgi?acc=GSE62327).

Field-specific reporting

Please select the one below that is the best fit for your research. If you are not sure, read the appropriate sections before making your selection.

- [x] Life sciences
- [ ] Behavioural & social sciences
- [ ] Ecological, evolutionary & environmental sciences

For a reference copy of the document with all sections, see nature.com/documents/nr-reporting-summary-flat.pdf

Life sciences study design

All studies must disclose on these points even when the disclosure is negative.

Sample size	No sample size calculation was performed for this study. In order to perform statistic analyses and achieve statistic significances, we mostly chose sample sizes of 3-6 for our in vitro experiments, and 6-8 for our in vivo experiments.
Data exclusions	Xenografts with sizes displaying more than twice the s.d. of the mean at the point of randomization were excluded for analysis, as tumors with outlying sizes might be derived from cancer cell clones that do not represent the cell line at a bulk cell level. Mice that died from unexpected illness along the experiment were excluded, as the measurement of tumor size could not continue. In the drug-tolerant cell assay, data derived from images that were out of focus were excluded, as those data are not accurate.
Replication	All in vitro experiments were repeated at least two times, unless stated otherwise. All attempts at replication were successful.
Randomization	The mice were randomized into four groups, when the tumors reached about 65 mm3. The randomization was conducted by equally dividing the tumor-bearing mice with similar tumor burden into each group.
Blinding	Quantification of IHC was performed by a person blinded to the treatment group. The qPCR analysis assessing PPP2R2B expression for the phase II clinical trial (ClinicalTrials.gov; Identifier: NCT01309607) was performed by a person blinded to the changes of the tumor sizes. Measurement of the tumor sizes was performed by a person blinded to the treatment groups.

Reporting for specific materials, systems and methods

We require information from authors about some types of materials, experimental systems and methods used in many studies. Here, indicate whether each material, system or method listed is relevant to your study. If you are not sure if a list item applies to your research, read the appropriate section before selecting a response.

Materials & experimental systems

n/a	Involved in the study	
[ ]	[x]	Antibodies
[ ]	[x]	Eukaryotic cell lines
[x]	[ ]	Palaeontology and archaeology
[ ]	[x]	Animals and other organisms
[ ]	[x]	Human research participants
[ ]	[x]	Clinical data
[x]	[ ]	Dual use research of concern

Methods

n/a	Involved in the study	
[x]	[ ]	ChIP-seq
[x]	[ ]	Flow cytometry
[x]	[ ]	MRI-based neuroimaging

Antibodies

Antibodies used	Primary antibodies: anti-p70S6K (cat. no. 9202), anti-phospho-p70S6K (Thr421/Ser424) (cat. no. 9204), anti-phospho-p70S6K (Thr389) (cat. no. 9234), anti-phospho-AKT (Ser473) (cat. no. 4058), anti-phospho-AKT (Thr308) (cat. no. 2965), anti-AKT (cat. no. 4691), anti-rpS6 (cat. no. 2217), anti-phospho-rpS6 (Ser235/236) (cat. no. 2211), anti-4EBP1 (cat. no. 9644), anti-phospho-4EBP1 (Ser65) (cat. no. 9451), anti-GAPDH (cat. no. 2118), anti-EZH2 (cat. no. 5246), anti-phospho-p44/42 MAPK (cat. no. 9101), anti-histone H3 (cat. no. 9715), anti-PP2A A subunit (cat. no. 2041), and anti-PP2A C subunit (cat. no. 2259) were purchased from Cell Signaling Technology. Anti-PPP2R2B (cat. no. ab16447) anti-H3K27me3 (cat no. 07-449) antibodies were purchased from Abcam and Merck Millipore, respectively; and anti-phospho-Myc (S62) (cat. no. 33A12E10) was acquired from BioAcademia. For detecting endogenous PPP2R2B, anti-PPP2R2B (LS-C761012) purchased from LSBio was used.<br>Horseradish peroxidase-linked secondary antibodies: anti-mouse IgG (NA931-1ML) and anti-Rabbit IgG (NA934-1ML) were acquired from GE Healthcare.<br>All primary antibodies were used in a dilution of 1:1000, expect anti-GAPDH used in 1:2500, and the secondary antibodies were used in 1:4000 dilution.
Validation	According to the manufacturer, there have been 76 citations reporting the reactivity to Homo sapiens for anti-p70S6K (cat. no. 9202) in western blotting, 19 for anti-phospho-p70S6K (Thr421/Ser424) (cat. no. 9204), 76 for anti-phospho-p70S6K (Thr389) (cat. no. 9234), 72 for anti-phospho-AKT (Ser473) (cat. no. 4058), 36 for anti-phospho-AKT (Thr308) (cat. no. 2965), 146 for anti-AKT (cat. no. 4691), 64 for anti-rpS6 (cat. no. 2217), 44 for anti-phospho-rpS6 (Ser235/236) (cat. no. 2211), 54 for anti-4EBP1 (cat. no. 9644), 14 for anti-phospho-4EBP1 (Ser65) (cat. no. 9451), 241 for anti-GAPDH (cat. no. 2118), 18 for anti-EZH2 (cat. no. 5246), 281 for anti-

phospho-p44/42 MAPK (cat. no. 9101), 35 for anti-histone H3 (cat. no. 9715), six for anti-PP2A A subunit (cat. no. 2041), and three for anti-PP2A C subunit (cat. no. 2259). Anti-PPP2R2B (cat. no. 16447) has been tested for western blot by Abcam, predicted to be reactive to Homo sapiens. Anti-H3K27me3 (cat no. 07-449) is a highly published Rabbit Polyclonal Antibody for western blot and reactive to Homo sapiens. Specificity of anti-phospho-Myc (S62) (cat. no. 33A12E10) has been validated by BioAcademia, and the antibody has be referenced by at least three citations used in western blotting and reactive to Homo sapiens. Specificity of anti-PPP2R2B (LS-C761012) has been shown by PPP2R2B knockdown experiment in this study (Fig. 3f).

Eukaryotic cell lines

Policy information about cell lines

Cell line source(s)	BT474, SKBR3, MB361, UACC812, and HEK293T were obtained from American Type Culture Collection (ATCC). BT474TR and BT474LR were derived from BT474 by culturing the cells at the presence of trastuzumab and lapatinib, respectively.
Authentication	The cell lines were routinely authenticated by growth curve analysis and morphology check using microscope.
Mycoplasma contamination	All cell lines were assessed regularly to ensure they were free of mycoplasma contamination.
Commonly misidentified lines (See ICLAC register)	No misidentified lines were used in this study.

Animals and other organisms

Policy information about studies involving animals; ARRIVE guidelines recommended for reporting animal research

Laboratory animals	Six-week-old female NCR nude mice maintained at 21°C ±1, in 55% to 70% humidity, and with a 12hr light/ dark cycle (from 7 am to 7 pm).
Wild animals	The study did not involve wild animals.
Field-collected samples	The study did not involve samples collected from the field.
Ethics oversight	All experimental or surgical protocols were conducted after receiving the approval from Institutional Animal Care and Use Committee of Singapore (IACUC), the Agency for Science, Technology, and Research (A*STAR).

Note that full information on the approval of the study protocol must also be provided in the manuscript.

Human research participants

Policy information about studies involving human research participants

Population characteristics	All patients are Asian female, aged ≥ 18 years, with histologic or cytologic diagnosis of breast carcinoma, and with T1-4 breast cancer with measurable primary breast tumor, defined as palpable tumor with the largest diameter measuring 2.0cm or greater by calipers. All patients are with tumors that are HER2 positive defined by either IHC (3+) or FISH amplification (amplification ratio >2.2). Patients must not have received prior chemotherapy or hormonal therapy for the treatment of breast cancer.
Recruitment	The trial is retrospective of the current study. Recruitment was performed based on the inclusion and exclusion criteria available on ClinicalTrials.gov (https://clinicaltrials.gov/ct2/show/NCT01309607). No self-selection bias was introduced in the recruitment.
Ethics oversight	The trial was approved by the National Healthcare Group Domain-Specific Ethics Review Board and Health Sciences Authority, Singapore.

Note that full information on the approval of the study protocol must also be provided in the manuscript.

Clinical data

Policy information about clinical studies

All manuscripts should comply with the ICMJEguidelines for publication of clinical research and a completedCONSORT checklist must be included with all submissions.

Clinical trial registration	NCT01309607
Study protocol	The full trial protocol can be accessed on ClinicalTrials.gov
Data collection	The patients were treated with neoadjuvant lapatinib plus chemotherapy (paclitaxel and carboplatin) for a total of four cycles. Tumor sizes were measured clinically before and after two or four cycles of treatment. The patients were enrolled between 2011 and 2013 in Singapore.
Outcomes	The primary endpoint was pathological complete response (pCR). Secondary endpoints were i) objective clinical response rates after 2 cycles of pre-operative treatment (i.e., complete or partial response according to RECIST v1.0), ii) rates of breast conserving surgery, iii) safety and tolerability, iv) disease-free and overall survival, and v) correlation of tumor IHC biomarkers and gene variants identified on next-generation sequencing with clinical outcomes.
